# A Solution to Chromium Toxicity? Unlocking the Multi-Faceted Role of Biochar

**DOI:** 10.3390/plants15020234

**Published:** 2026-01-12

**Authors:** Muhammad Umair Hassan, Qitao Su

**Affiliations:** Key Laboratory of Jiangxi Province for Biological Invasion and Biosecurity, School of Life Sciences, Jinggangshan University, Ji’an 343009, China; muhassanuaf@gmail.com

**Keywords:** biochar, chromium, human health, immobilization, soil health

## Abstract

Chromium (Cr) toxicity poses a significant challenge to agricultural productivity, human health, and food security. Biochar (BC) is a versatile amendment employed to alleviate Cr toxicity. Chromium stress impairs growth by inducing membrane damage and cellular oxidation, as well as inhibiting chlorophyll synthesis, photosynthetic efficiency, water uptake, and nutrient absorption. This review consolidates information on the mechanisms through which BC mitigates Cr stress. Biochar facilitates Cr immobilization by reduction, adsorption, precipitation, and complexation processes. It enhances growth by improving photosynthetic efficiency, water and nutrient uptake, osmolyte synthesis, and hormonal balance. Additionally, biochar promotes resilient bacterial communities that reduce Cr and enhance nutrient cycling. The effectiveness of BC is not universal and largely depends on its feedstock properties and pyrolysis temperature. This review provides insights into soil quality, plant function, and human health, which contribute to providing a comprehensive assessment of the capacity of BC to mitigate Cr toxicity. This review highlights that BC application can reduce Cr entry into the food chain, thus decreasing its health risk. This review also identifies knowledge gaps and outlines future research directions to increase the efficiency of BC in mitigating Cr toxicity. This review also offers insights into the development of eco-friendly measures to remediate Cr-polluted soils.

## 1. Introduction

Soil heavy metal (HM) pollution is becoming a serious challenge for safer food productivity [1,2]. These metals persist in the soil for extended periods, leading to the degradation of soil quality and adverse effects on plant health [3]. Chromium (Cr) is recognized as a toxic metal that poses a substantial threat to human health [4,5]. Chromium occurs in two oxidation states, trivalent chromium (Cr-III) and hexavalent chromium (Cr-VI), with the latter being more mobile, toxic, and bioavailable [6]. The presence of chromium adversely affects plant growth, development, physiology, morphology, and gene expression [7]. Chromium toxicity impairs seed germination, root and shoot growth, chlorophyll synthesis, enzyme activity, and nutrient uptake, ultimately resulting in reduced plant growth [8]. Additionally, Cr exposure diminishes photosynthesis, water use efficiency (WUE), and hormonal balance while increasing the production of reactive oxygen species (ROS), which leads to the oxidation of DNA, proteins, and lipids [9].

Plants absorb chromium (Cr) through their roots and subsequently translocate it to shoots and leaves. Elevated Cr concentrations in plant tissues adversely affect the development of floral organs and fruits, thereby compromising crop quality [10]. Additionally, chromium impairs nutrient and water uptake and disrupts antioxidant defenses, resulting in diminished growth, yield, and quality [11]. Despite these challenges, plants have evolved various strategies to mitigate Cr toxicity. For example, they enhance their antioxidant defense systems and sequester Cr in vacuoles to reduce its harmful effects [12]. However, these protective mechanisms vary in effectiveness across different plant species [13]. Furthermore, plants modulate gene expression and increase the synthesis of solutes and osmolytes to combat Cr toxicity [14,15].

Various strategies, such as phytoremediation and chemical stabilization, are being implemented worldwide to protect crops and human health from the detrimental effects of Cr [16]. These methods are often expensive, unsustainable, and less effective, highlighting the need for more cost-effective and efficient techniques to address Cr toxicity [17]. In this context, biochar has emerged as a promising solution for mitigating the toxicity of HMs [18,19]. Biochar, a carbon-rich material produced through the pyrolysis of wood, crop residues, and various waste materials [20,21], enhances soil fertility, nutrient availability, water retention capacity, soil structure, and Cr adsorption, facilitating its transformation into less toxic forms [22,23]. Additionally, biochar enhances antioxidant activities and improves plant physiological and molecular functions, thereby promoting better growth in Cr-contaminated soils [24]. The efficacy of biochar in mitigating heavy metal and metalloid toxicity is largely influenced by the properties of the biochar and soil, as well as the concentration of toxic metals/metalloids present [25]. For example, BC produced at lower temperatures may exhibit elevated concentrations of volatile compounds, polycyclic aromatic hydrocarbons (PAHs) and toxic metals [26,27]. The presence of toxic metals in biochar can enhance their availability and accumulation in plants, potentially leading to reduced plant growth [28]. Moreover, the existence of these toxic substances may exert additional ecotoxicological effects, thereby limiting the efficacy of biochar in remediating polluted soils. Consequently, careful consideration must be given to the selection of feedstock for BC production. Current studies highlight the potential of BC to remediate Cr-contaminated soils. Nevertheless, a comprehensive and critical synthesis of interconnected mechanisms involved is still lacking. This review aims to elucidate the effects of BC on Cr immobilization and the mitigation of its toxic impacts through various mechanisms. It seeks to provide insights for optimizing biochar production to achieve remediation objectives. Furthermore, this review identifies existing knowledge gaps and proposes a roadmap for translating scientific insights into strategies for sustainable food production and the remediation of Cr-polluted soils.

## 2. Mechanisms of Chromium Uptake and Its Toxic Impacts on Plants

Chromium presents a significant environmental challenge due to the release of high concentrations of Cr from agricultural and industrial activities [29]. The maximum permissible concentration of Crin soils is 64 mg kg^−1^, and exceeding this limit can have detrimental effects on both plants and humans. It has a wide range of uses including cement, steel, leather, metal plating, paper, and timber production, contributing to its environmental presence [30]. Furthermore, flash outs from the coal, municipal, and fertilizer industries are also responsible for the entry of Cr into the environment [31]. Naturally, Cr enters soils and water from volcanic dust and rocks [32]. Plants take up Cr via different transporters; for example, Cr-VI in plants is transported by ion transporters such as those involved in sulfate transport [33]. Chromium competes with iron (Fe), phosphorus (P), and sulfur (S) for carrier binding during transportation [34]. Due to it structural similarity to sulfate and phosphate, Cr-VI is absorbed by plants via sulfate and phosphate transporters [35,36]. Sulfur accumulator plants such as Brassica plants absorb relatively high levels of Cr [37]. This suggests that the mechanism of S uptake and transport may involve the movement of Cr from the roots to the shoots [38]. Different meal transport families, such as *HMA*, *ABC*, *CDF*, *NRAMP*, and *ZIP*, play critical roles in the transport of metals from roots to shoots [39]. Nevertheless, the role of these metal transport families in Cr absorption, transport, and sequestration has not been fully explored.

**Figure 1:**
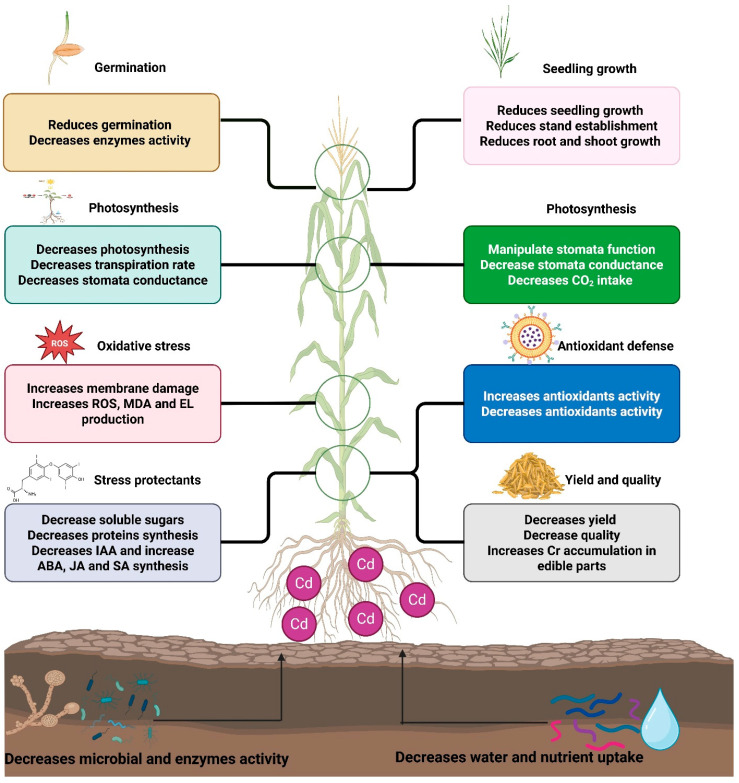
Toxic effects of chromium on plants. Chromium increases reactive oxygen species (ROS), membrane damage, and stress hormones and decreases nutrient and water uptake, thereby causing growth and yield losses.

Chromium toxicity adversely affects plant physiological functions, leading to reduced seed germination, stunted growth, and diminished final yield [40]. The inhibition of seed germination due to Cr has been extensively documented in various crops, including cauliflower, wheat, barley, and maize. Photosynthesis, a critical process in plants, is negatively impacted by Cr toxicity, which reduces chlorophyll synthesis (Figure 1) and damages the photosynthetic apparatus [39]. Furthermore, chromium induces phytotoxicity in both soil and plant systems by adversely affecting nutrient absorption, transport, and distribution [41]. The structural similarities between Cr and essential nutrients result in complex alterations in plant mineral nutrition. Both forms of Cr have been reported to interfere with nutrient uptake, consequently impairing plant growth [42].

Chromium accumulation in plants adversely impacts water absorption, resulting in diminished water content within plant cells [43]. It impairs root growth and water uptake (Table 1) and reduces water (Figure 1) absorption by plants, leading to a reduction in plant growth [44]. Furthermore, Cr-mediated structural changes also decrease the ability of plants to obtain water from soil [45]. This decline in water uptake and availability reduces seed germination and leads to a reduction in plant growth [39]. Chromium toxicity increases ROS production, causing the oxidation of proteins, lipids, and cellular membranes [46]. The excessive generation of ROS also disrupts cellular homeostasis, impairs membrane function, affects photosynthetic pigments and degrades genetic materials [47]. Chromium exposure also increases the concentrations of ascorbic acid (AsA) and glutathione (GSH) and decreases the concentrations of phenolic compounds [48].

Chromium toxicity also negatively affects different metabolic processes, such as electron transport, carbon dioxide (CO_2_) fixation, enzyme activity, and photophosphorylation, which impair photosynthesis [68]. Chromium also destroys the photosynthetic apparatus, which is the harvesting complex of PS-I and PS-II, and prevents the production of enzymes involved in the Calvin cycle [69]. It also reduces the level of net photosynthesis, chlorophyll synthesis, water use efficiency (WUE), transpiration, and stomatal conductance [70]. Moreover, Cr-mediated degradation of the photosynthetic apparatus also causes a reduction in light harvesting [71]. Nevertheless, the severity of Cr stress largely depends on its concentration, and it has damaging effects in a dose-dependent manner. For example, Alharby and Ali [72] tested the impacts of various Cr concentrations (50 and 100 mg kg^−1^) on rice plants. They reported that 100 mg kg^−1^ significantly reduced root and shoot growth, leaf area, chlorophyll synthesis, and gas exchange properties and increased the production of EL, MDA, and ROS. A recent study by Al-Huqail et al. [73] reported that the dose (75 and 150 mg/L) of Cr had a dependent effect on rice plants. The highest level of Cr stress (150 mg L^−1^) decreased plant growth and biomass production, photosynthetic pigments, soluble sugars, and nutrient availability. A study conducted on wheat plants also revealed the dose (50, 100, and 200 mg kg^−1^)-dependent effects of Cr [73]. Higher doses of Cr caused a marked reduction in biomass, carotenoids, leaf water contents, stomatal conductance, and photosynthetic and transpiration rates while increasing oxidative damage and ROS production [74]. Likewise Rafique and colleagues studied the impacts of different rates of Cr-VI (5, 10, 20, 40 mg L^−1^). They reported that the maximum Cr concentration (40 mg L^−1^) caused a considerable decrease in growth traits. Plants modulate gene expression levels to counteract Cr stress. A study conducted in Brassica showed that the expression levels of genes involved in Cr-vacuolar sequestration increased in plants, which helped counteract Cr toxicity [75]. Similarly, Jain et al. (2016) reported that the expression of the metallothionein (*MT*) gene increased in leaves and stems under Cr stress [76]. Furthermore, Gill et al. (2016) reported a substantial increase in the expression levels of the *BnaA08g16610D*, *BnaCnng19320D*, and *BnaA08g00390D* genes in response to Cr, contributing to an increase in Cr tolerance [77]. In addition, Colzi et al. [78] reported that, compared with F0 plants, F1 Arabidopsis plants promptly activated genes involved in Cr stress responses under relatively low Cr stress. They also noted that under relatively high Cr stress, F1 plants modulated fewer genes than did F0 plants. Moreover, many *bHLH* transcription factors are induced by Cr stress in F1 plants but not in F0 plants [78]. Soil bacterial diversity and gene expression are also significantly affected by Cr stress. A recent study revealed that under Cr stress, bacterial genera such as *Sphingomonas* were upregulated, whereas the activity of Cr-VI reductase genes such as *chrR* and *nfsA* was upregulated. This reshaping of soil microbes’ upregulated the expression of specific sugars, amino acids, and ABC transporters, which helped in Cr reduction and detoxification [79]. Chromium stress also affects root exudates, which play a crucial role in Cr tolerance. Root exudates (citric acid) contribute to the reduction and immobilization of Cr by driving the soil iron and sulfur cycles. Another study reported that root exudates and citric acid decreased the soil pH, increased the soil organic matter content, and created favorable conditions that facilitated the reduction of Cr-VI into extractable, oxidizable, and residual forms of Cr [80].

## 3. Role of Biochar in Mitigating Chromium Toxicity

Biochar has shown appreciable results in mitigating Cr toxicity in soil and plant systems. Biochar modulates plant physiological and biochemical functions, improves nutrient and water uptake, and increases soil carbon availability. It also increases Cr immobilization and microbial activity and decreases the availability of Cr, thus ensuring better growth in Cr-polluted soils. The details of the different mechanisms by which BC mitigates Cr toxicity are explained below.

### 3.1. Biochar Improves Leaf Water Status and Maintains Membrane Integrity in Response to Chromium Stress

Biochar protects the cellular membrane and maintains the water status to counter Cr toxicity. The literature has demonstrated that Cr toxicity affects plant water relationships and membrane integrity through oxidative damage. Recent studies have shown a clear trend of Cr being involved in RWC and an increase in EL. For example, Bashir et al. [81] reported a decrease of 12% in RWC and an increase of 12.1% in EL under Cr stress, whereas Sami et al. [82] reported a more disruptive effect, with a decrease of 11.86% in RWC and a 64.03% increase in EL. The increase in EL was linked to oxidative stress, where Cr increased ROS production, which in turn increased MDA production, leading to a reduction in membrane permeability. Biochar has emerged as an effective strategy to maintain water relations and mitigate EL. Biochar reverses the symptoms of Cr and enhances membrane stability by increasing antioxidant activity, proline synthesis, and soil nutrient availability and decreasing MDA production [82]. This was evidenced by recent discoveries showing that BC caused a concurrent reduction in EL and an increase in RWC (20.13–23.5%) [81,82,83]. These studies assessed the different types of BC; therefore, results may vary with BC made from different feedstock under different pyrolysis temperature. These were the short-term studies performed in controlled conditions, which suggest performing field studies. The restoration of membrane integrity is further affected by the membrane stability index (MSI). Chromium stress reportedly decreases the MSI by 99% [84], whereas BC reportedly increases the MSI. This protective effect of BC is associated with its dual actions. First, it increases defense responses such as proline synthesis and antioxidant activities, which protect cellular membranes. Second, BC also increases soil nutrient availability, which favors plant function, therefore maintaining a better MSI. For example, Mazhar et al. [85] reported that BC application increased MSI by increasing proline synthesis, which provides protection to plants against stress.

MDA is produced as a result of lipid damage caused by ROS, and it is an important indicator of oxidative stress. The application of BC increases antioxidant activity, which reduces MDA production owing to a reduction in the oxidation of lipids [86]. The increase in ROS production damages the cellular membrane and leads to the loss of electrolytes, resulting in an increase in EL [87]. A recent study by Dong et al. [88] revealed that BC applied to Cr-polluted soil reduced hydrogen peroxide (H_2_O_2_) in the roots and shoots of *Cyamopsis tetragonoloba* by 24.76 % and 39.01 %, respectively. They also reported that the same treatment decreased the MDA content in shoots and roots by 45.03% and 34.84% and the EL content in shoots and roots by 13.93% and 9.83%, respectively [88]. This decrease in oxidative stress biomarkers was associated with increased antioxidant activity and proline synthesis and decreased Cr accumulation.

**Figure 2:**
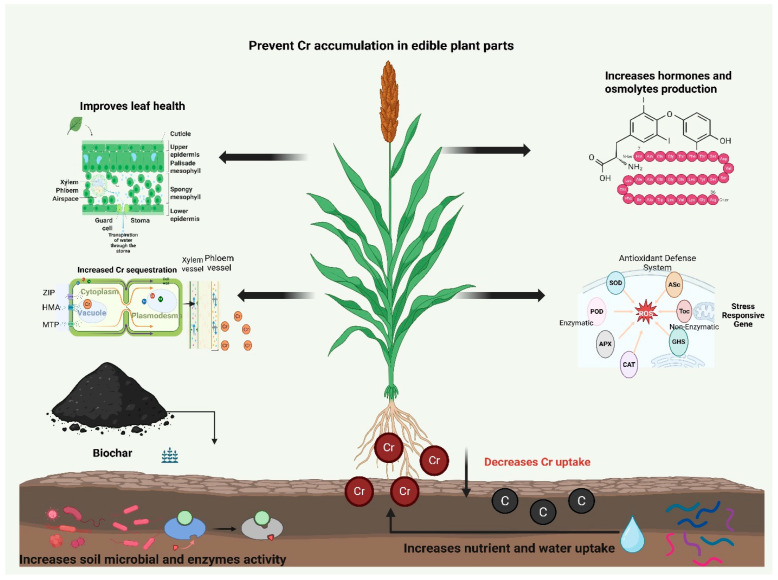
Mechanisms by which biochar mitigates chromium toxicity in plants. Biochar increases soil microbial activity, nutrient water availability, antioxidant activity, and photosynthetic efficiency and decreases membrane damage, reactive oxygen species (ROS) production and the synthesis of stress-responsive hormones, thus increasing growth under Cr stress conditions.

In summary, BC confers physiological resilience in plants by decreasing oxidative damage. The primary mechanism involved is the reduction in ROS, MDA, and EL production (Table 2). These findings indicate that BC indirectly reduces Cr availability and accumulation, thereby decreasing ROS production. Second, BC also has a direct biochemical effect on plants, as indicated by the robust increase in antioxidant activity and osmolyte production (Figure 2). Moreover, BC improved the soil WHC, thereby maintaining better leaf RWC in Cr-contaminated soil. There is a positive association between reduced Cr uptake and oxidative damage; nevertheless, the specific signaling mechanism through which BC augments antioxidant genes has not yet been reported. Furthermore, most of these studies explored physiological and biochemical mechanisms; therefore, a deeper molecular understanding is needed to explore these mechanisms.

### 3.2. Biochar Improves Photosynthetic Efficiency Under Chromium Stress

Photosynthesis is an important process in plants and plays a crucial role in energy production. Chromium impairs photosynthesis by causing deficiencies in nutrients such as N and Mg and triggering oxidative damage [106]. Biochar counteracts these effects by immobilizing Cr and reducing its availability and uptake by plants [107,108]. This reduction in Cr uptake also improves nutrient uptake by roots, which helps to restore chlorophyll synthesis and subsequent photosynthetic efficiency [83,88]. For example, the application of BC has been reported to increase photosynthetic efficiency, chlorophyll synthesis (Figure 2), and transpiration rates by increasing soil nutrient availability and antioxidant activities and decreasing Cr availability [82,109]. However, the BC-mediated increase in the aforementioned traits depends on the plant species and dose of BC. The recovery of photosynthetic pigments is associated with gas exchange traits. For example, Naveed et al. [109] reported that BC increased the photosynthetic and transpiration rates, stomatal conductance, and RWC by 151, 104, 127, and 102%, respectively, in Cr-polluted soil. Bashir et al. [81] reported that 3% BC enhanced total Chl-a, Chl-b, and carotenoid (Cart) contents; transpiration; the photosynthetic rate; and stomatal conductance by 21.5%, 21.4%, 19.8%, 18.8%, 20.3%, 24.8% and 19.2%, respectively, in maize grown in Cr-polluted soil [110]. Another recent study revealed that BC application (0.1, 0.5, 1, and 3 g L^−1^) increased photosynthetic pigments in a dependent manner. Notably, 3 g L^−1^ BC application considerably increased Chl-a (167.55%), Chl-b (68.97%), total Chl (128.47%), and Cart (183.33%) synthesis compared with the other rates. These findings indicate that high concentrations of BC increase photosynthetic pigments and overall photosynthetic efficiency. However, it largely depends on biochar and soil properties and the severity of stress conditions. Biochar-mediated improvements in soil properties and reductions in Cr availability support photosynthetic traits, leading to better growth under Cr stress [82,110]. A biochar-mediated increase in stomatal conductance favors greater transpiration and CO_2_ assimilation, which in turn increases the photosynthetic rate and subsequent dry matter production [109,111]. However, these mechanisms are further strengthened by the synergistic application of BC with other amendments. For example, Li et al. [112] reported that BC combined with arbuscular mycorrhizal fungi (AMF) significantly increased the photosynthetic rate and overall photosynthetic efficiency. This was linked with increased nutrient and water availability and reduced Cr translocation [112]. This synergy highlights the importance of the BC-soil and microbe interfaces in improving plant function under Cr stress. These studies also identified various research gaps. For example, BC induced a dose-dependent effect, and BC at 3–5% was particularly effective at improving photosynthetic efficiency. All of these findings are from laboratory experiments, and the long-term stability of photosynthetic improvement must be validated under field conditions. Moreover, the effects of BC properties, particularly pyrolysis temperature and feedstock type, on photosynthesis have not yet been defined.

### 3.3. Biochar Improves Osmolyte Synthesis, Hormonal Balance and Antioxidant Defense in Response to Chromium Stress

Different osmolytes, including sugars, proline, and glycine-betaine (GB), play crucial roles in counteracting Cr toxicity [113,114]. Chromium stress decreases the synthesis of soluble sugars and proteins by decreasing soil nutrient availability [88]. Chromium stress inhibits the availability of essential nutrients such as N, leading to a decrease in protein and sugar synthesis [115]. For example, in guar plants, Cr decreased the soluble sugar content in the shoots and roots of guars by 40.49% and 45.55%, whereas the soluble protein content in the shoots and roots decreased by 13.31% and 31.78%, respectively [88]. This depletion compromises energy reserves and plant function, leading to a reduction in plant growth [116]. Biochar mitigates Cr toxicity by restoring the synthesis of proteins and sugars, which play crucial roles in stress tolerance. For example, Dong et al. (2025) reported that BC under Cr stress increased sugar and protein synthesis by 70.50 % and 41.38 %, respectively [88]. This increase was associated with increased phloem activity, which triggers cell division and leads to an increase in protein and soluble sugars [117]. Furthermore, BC also optimizes resource allocation and improves protein metabolism, hence increasing protein synthesis [91]. Thus, through these mechanisms, BC maintains metabolic homeostasis, thus enhancing growth and Cr resilience. These studies were conducted in controlled systems, which show the effectiveness of BC in improving plant functions; however, these results can vary under real-field conditions.

Proline plays a critical role in the adaptation of stressed plants to stress by maintaining osmotic adjustments, antioxidant activities, and cellular stabilization. Proline synthesis is increased under Cr stress, which is an important physiological response used by plants to counteract abiotic stress-mediated oxidative damage [118]. Biochar has been reported to decrease proline synthesis by immobilizing Cr and reducing its toxicity and oxidative stress in plants. Biochar can alleviate stress effects; therefore, the demands of plants for osmotic adjustment via proline synthesis decrease. Therefore, different authors reported a significant decrease in proline synthesis in BC-amended plants grown in Cr-polluted soils [91]. Hormonal signaling plays a critical role in plants to withstand stressful conditions. Plants increase the synthesis of abscisic acid (ABA), jasmonic acid (JA), and salicylic acid (SA), which regulate different adaptation and defense pathways [119,120]. Biochar application modulates hormonal shifts, and this relationship occurs via primary and secondary mechanisms. The primary indirect mechanism is the immobilization of Cr by BC, which reduces its uptake by plants. This, in turn, allows the plants to maintain the hormonal balance, as previous studies reported that BC decreased the synthesis of stress-related hormones such as ABA, JA, and SA [121]. The secondary direct mechanism is associated with improved soil fertility and the availability of nutrients, such as nitrogen and potassium. The improved nutrient availability supports metabolic pathways such as tryptophan synthesis. This, in turn, increases IAA production, thereby promoting plant growth under stress conditions [120,121]. Therefore, these findings support the indirect and direct pathways by which BC maintains hormonal balance; however, future studies are needed for targeted research.

Biochar alleviates Cr toxicity by decreasing its availability, which modulates plant antioxidant defense and helps counteract Cr toxicity. The primary mechanism involved is the immobilization of Cr and its subsequent uptake and ROS production. This action enhances the antioxidant defense and normalizes the antioxidant activity to a control level. Therefore, the frame explains the apparent contradictions in the literature. Different studies reported a significant increase in response to Cr stress after BC application. Research on different crops, including brassica and maize, has shown that BC enhances antioxidant activity by decreasing Cr uptake [109,122]. Dai et al. [123] also reported that CAT and POD activities were increased under Cr stress and with the application of BC. However, there was no difference between the control and BC-treated soils, indicating that BC application completely reduced Cr-induced oxidative damage [123]. Recently, Shahzad et al. [124] reported that BC enhanced APX, CAT, POD, and SOD activities in Cr-polluted soils and mitigated the adverse impacts of Cr-induced oxidative damage. Different authors also reported that CAT activity increased by 50.6% with the application of BC in Cr-polluted soil, whereas SOD activity increased by 23%, which provided a defense to plants against Cr stress [125,126]. Recently, Dong et al. [88] reported that BC treatment increased SOD activity in guar shoots and roots by 11.84% and 6.22%, respectively; furthermore, BC also increased POD and CAT activities. Biochar rapidly reacts with Cr, thus decreasing its availability, which enables the plants to scavenge Cr from their body by increasing their antioxidant activity [88]. The elevated level of antioxidant activity directly scavenges ROS, reduces membrane damage and lipid peroxidation, and supports plant growth. This efficiency can be further amplified by using BC modification techniques and the integrated use of BC with other amendments. For example, zinc-modified BC mitigated the direct toxic impacts of Cr by increasing ascorbate peroxidase (APX) and POD activities [86]. Moreover, compared with their application alone, BC in combination with PGPR substantially increased APX, CAT, and SOD activities in *S. oleracea* seedlings in Cr-polluted soil [127]. Conversely, some studies reported that BC decreased the levels of antioxidants. When BC effectively immobilizes Cr to background levels, the oxidative stress is removed. Therefore, under such conditions, an increase in antioxidant defense diminishes, and the activity of enzymes returns to the level of unstressed plants. This explains the findings where BC significantly decreased antioxidant activity, proline synthesis, and lipid peroxidation [110,123]. The literature shows that BC clearly enhances the antioxidant activity to quench the reduced oxidative stress caused by a lower concentration of Cr. In some studies, where BC effectively immobilized Cr, the antioxidant activity returned to control levels owing to a substantial reduction in oxidative damage. This finding explains why some studies reported an increase in antioxidant activity, whereas others reported a decrease in antioxidant activity. Furthermore, signaling pathways that connect metal uptake to the downregulation of antioxidants have not yet been studied. Future work should focus on these aspects to increase the understanding of the role of BC in mitigating Cr toxicity.

### 3.4. Biochar Improves Nutrient Uptake and Accumulation in Plant Parts in Response to Chromium Stress

Nutrients play crucial roles in plant growth and tolerance to heavy metals. Chromium stress disturbs nutrient uptake, thereby causing growth losses [25]. However, BC increases nutrient uptake and helps counteract Cr toxicity. The primary mechanism is Cr immobilization, which reduces the competition between Cr and nutrients, thereby increasing nutrient uptake and accumulation [128,129]. Recently, BC was shown to increase N, P, K, Ca, and Mg accumulation in guar plants by 18.32 %, 22.91 %, 23.89 %, 37.84 % and 20.81 %, respectively [88]. This was linked with the ability of BC to adsorb Cr ions, hence reducing competition with nutrients, thereby increasing nutrient accumulation [130]. A critical synthesis from the literature shows that BC has a dose-dependent effect on nutrient uptake. For example, Qin et al. [131] reported that Cr toxicity decreased nutrient accumulation in Chinese cabbage owing to intense competition with nutrients. They reported that BC (0, 0.1, 0.5, 1, and 3 g/L) increased the N, P, K, Ca, and Mg concentrations in Chinese cabbage. They reported that BC concentrations ranging from 0.1 to 3 g L^−1^ tended to increase with increasing nutrient concentration. Research by Yue et al. [90] revealed that BC (0.5, 1.25, and 2.5 L^−1^) had a dose-dependent effect on increasing nutrient concentrations in plants [90]. They reported that 2.5 g L^−1^ BC application increased the N (20.82%), P (37.11%), K (24.95%), Ca (6.14%), and Mg (22.33%) concentrations in plants facing Cr stress [90]. This recovery process is associated with the ability of BC to adsorb Cr, reduce its availability, and improve plant function and subsequent nutrient uptake [132,133]. Recently, Li and colleagues reported that Cr toxicity reduced root and shoot NPK uptake, and the most profound decrease was observed in K uptake by roots and shoots. In contrast, the application of BC, particularly 1% and 5%, increased NPK uptake by the plants. The application of BC (1 and 5%) increased K accumulation in roots and shoots by 1.6–2.1 and 21.8–23.4 times, respectively. Siddika et al. [134] studied the impact of rice stubble and sawdust BC produced at 450 °C and modified it with KOH (1 M). They applied 20 t ha^−1^ BC to Cr-polluted soil (0, 100, 200, and 300 µg g^−1^) and reported that BC and KOH-modified BC increased the soil NPK, EC, and cation exchange capacity (CEC) [134]. These findings indicate that the rate of BC application must be considered to increase its efficiency against Cr toxicity. Nevertheless, the effects of BC can affect the soil properties, BC type, and severity of Cr stress. Biochar also supports plant growth by improving soil physicochemical properties. Its application increases soil organic carbon (SOC), water holding capacity, and soil structure, hence creating favorable conditions for nutrient uptake [135,136]. The increase in SOC caused by BC favors microbial activity and nutrient release, contributing to better growth and development under Cr stress [136]. These findings suggest that the BC-mediated increase in nutrient concentration is a twofold process. BC absorbs Cr, reduces its availability, and decreases the degree of competition between nutrients and Cr. This, in turn, increases nutrient uptake and subsequently increases nutrient accumulation. Second, BC works as a slow-release fertilizer, and it directly increases the soil nutrient pool. This synergy between these two pathways increases nutrient availability and ensures better growth recovery in Cr-polluted soils. The present literature is also phenomenological, which shows that BC improves nutrient uptake. However, there are gaps in knowledge concerning the physiological and molecular mechanisms by which BC enhances nutrient uptake. For example, it is unknown whether BC upregulates the expression of nutrient transporter genes or whether it improves nutrient accumulation by reducing the competition between nutrients and Cr. Additionally, the long-term effects of BC on nutrient cycling in Cr-polluted soils are not yet understood.

### 3.5. Biochar Increases Chromium Immobilization and Reduces Its Uptake to Counter Chromium Stress

Biochar reduces Cr availability through interconnected mechanisms such as adsorption, reduction, and pH-mediated immobilization [112,137]. Biochar has a relatively high porosity and a large surface area, which provides sites for Cr adsorption and therefore helps in removing Cr from the medium [138]. An increase in soil pH increases Cr immobilization, which therefore reduces Cr availability and decreases Cr uptake by plants [139]. Biochar also facilitates the reduction of Cr-VI into the less toxic form Cr-III by electron transfer from functional groups and solubilization of the constituents of BC [139,140]. The functional groups present on BC can transfer the forms of Cr by modulating the soil pH [141]. Biochar also fixes Cr and adsorbs Cr on its surface, which prevents its availability and uptake by plants [23,142]. The efficiency of these mechanisms largely depends on the BC properties. For example, Rafique et al. [143] reported that BC made at different temperatures (300, 500, and 700 °C) decreased Cr availability by 45.5%, 25.5% and 32.8%, respectively. The increase in Cr removal at lower temperatures was associated with the presence of more functional groups along with organic and mineral components [144]. Sehrish et al. [145] noted that BC (5%) made from poultry litter reduced Cr availability by 51.5%. Furthermore, modification of BC also affects the ability of BC to decrease Cr availability. For example, Shan et al. [146] modified BC with hydrochloric acid (HCl), potassium hydroxide (KOH), and zinc chloride (ZnCl_2_) and reported that ZnCl_2_ modification effectively decreased Cr from polluted media via ion exchange, complexation, and electrostatic interactions. Different studies have shown that acid-modified BC is an effective tool for remediating Cr-VI in soil by means of pore filling, sorption, and reduction [147]. Recently, Cao et al. [148] reported that BC rice straw, corn straw, bamboo and wood decreased Cd uptake and accumulation via modifying soil properties and reshaping soil microbial community. Moreover, Su et al. [149] reported that BC modification with nZVI enhanced Cr immobilization by 92.9%. Chen et al. [150] used Fe-modified BC to immobilize Cr in soil and water. They reported that the application of Fe-modified BC reduced Cr availability from groundwater by 71%. Furthermore, BC immobilizes Cr in topsoil from both soil and water and decreases Cr-VI leachability by 86%. These findings show that BC causes Cr immobilization through a set of mechanisms that can be divided into direct and indirect pathways. The indirect pathways are mediated by an increase in soil pH, which augments the reduction of Cr-VI into Cr-III. The direct mechanism involves the presence of functional groups on the BC surface, which work as electron donors, hence reducing Cr-VI to Cr-III. This group also favors Cr complexation with organic substances present in BC, thus decreasing its availability. Nevertheless, most of these studies were performed in pots, and the long-term stability of Cr complexes has not been determined. Furthermore, modified BC is a promising technique; nevertheless, secondary contamination at the field scale could be a barrier to its practical use. Moreover, many studies have correlated the decrease in Cr availability with increased pH; however, differentiating this effect from adsorption and reduction processes is difficult. Future studies should explore the long-term stability of Cr complexes under field conditions.

### 3.6. Biochar Improves Soil Biological Properties and Microbial Activity to Counter Chromium Stress

Soil enzymes play crucial roles in nutrient cycling in polluted soils [151]. Heavy metals disturb soil enzyme activity, whereas BC results in increased soil enzyme activity. For example, Guo et al. [152] reported a significant increase (29.06%) in urease activity in Cr-polluted soil after BC application. Furthermore, Yang et al. [151] reported that, compared with the control, BC addition significantly increased phosphatase and sucrase activity in Cr-polluted soils [151]. Nevertheless, Huang et al. [153] reported that straw-based BC inhibited alkaline phosphatase, invertase, and urease activities in Cr-polluted soil during a 30 30-day incubation study [153]. Liu et al. [154] reported that the activity of glucosidase and phosphomonoesterase decreased over time; however, this decrease was reduced in BC treatments. These findings indicate that the BC structure and properties determine the complex impacts on soil enzyme activity. For example, adsorption of the reaction substrate by BC improves the enzymatic reaction and activity. Therefore, the soil properties, BC rate, and properties can affect soil enzyme activity [153].

Toxic metals negatively affect soil microbial activity, whereas BC counters the toxic impacts of Cr on soil microbes by causing Cr immobilization. Biochar adsorbs and stabilizes Cr, particularly converting Cr-IV into Cr-III, thereby reducing the toxicity of Cr to soil microbes [155,156]. This alleviation of Cr stress restored the soil microbial abundance, diversity, and community structure. Recent findings have shown that BC application to Cr-polluted soils enhances operational taxonomic units (OTUs) and alpha diversity indices (Chao, Ace, and Shannon), resulting in substantial increases in microbial richness and diversity [157,158]. In particular, BC has been reported to increase the abundance of *Actinobacteria* and *Firmicutes* bacteria, which possess excellent metal resistance properties [159,160]. The increase in the abundance of Firmicutes is linked with Cr immobilization in soil [160]. The bacterial phylum often possesses genera with robust chromate reductase genes and excellent efflux systems, which ensure the direct enzymatic reduction of Cr-VI and its cellular detoxification [161]. Similarly, Actinobacteria also promote metal sequestration by producing biofilms and siderophore production and drive the metabolic pathways involved in stress survival [162]. Moreover, BC also reshaped soil bacterial communities by widening ecological niches and decreasing competitive overlap, thus promoting a resilient ecosystem [102]. This change leads to improved microbial functioning, improved soil health, and functional genes linked with Cr resistance and reduction. The biochar-mediated increase in microbial activity is associated with two mechanisms. First, BC increases the abundance of core bacteria involved in energy, carbohydrate and amino acid metabolism, which are suppressed under Cr toxicity [102]. Second, it is also associated with increased enzyme activity, which is a direct indicator of microbial metabolic function [163]. Enzymes such as dehydrogenases and oxidoreductases play crucial roles in microbial redox and respiration reactions and Cr reduction reactions. The provision of stable carbon and habitat by BC improves the aforementioned processes, hence increasing the overall microbial resilience in Cr-polluted soils [164]. This effect is further increased with synergistic applications such as a combination of BC and AMF, which promotes bacterial diversity and evenness [102]. These findings suggest that BC generally enhances microbial abundance; nevertheless, its impacts on soil enzyme activity are nuanced and contingent on specific conditions. Biochar primarily increases the abundance of soil microbes by increasing Cr adsorption and bioavailability. The adsorption of Cr prevents enzyme denaturation and stimulates microbial activity. Nevertheless, this potential can vary on the basis of the soil properties, biochar properties, study duration, and enzyme type. These findings also highlight that the long-term fates of enzymes sequestered on the BC surface are unknown. Furthermore, BC reshapes soil bacterial communities; therefore, the role of gene expression linked with Cr resistance and nutrient acquisition must be explored in new microbial communities.

### 3.7. Biochar Improves Growth and Yield and Decreases the Health Risk of Growing Crops in Chromium-Polluted Soils

Chromium stress negatively affects plant growth by causing oxidative damage and disturbing plant function. Biochar mitigates Cr toxicity to plant growth through a series of interconnected actions. Biochar increases Cr immobilization and decreases availability, and this reduction leads to better nutrient homeostasis, antioxidant defense, and osmolyte synthesis; these changes collectively improve plant growth. For example, Deng et al. (2024) reported that BC (5 and 10%) significantly enhanced wheat growth in a dose-dependent manner under Cr stress (50–200 mg kg^−1^), which stemmed from improved nutrient availability, SOC, and antioxidant availability and reduced Cr accumulation in plant parts [74,165]. Similarly, other researchers reported that BC enhanced growth and biomass (60–200%) by decreasing Cr uptake and accumulation and increasing nutrient uptake and plant antioxidant activity [81,126]. Fan et al. [166] reported a substantial increase in germination and growth in spinach with BC. The literature also shows that BC has a dose-dependent effect on improving plant growth under Cr stress. For example, Yue et al. (2025) reported a dose-dependent response of BC (0.5, 1.25, and 2.5 g L^−1^) in increasing cabbage growth and yield, which was associated with improved nutrient uptake and antioxidant activity and reduced Cr uptake and accumulation [90]. The properties of biochar also influence its ability to affect plant growth under Cr stress conditions. Alami-Milani et al. [101] observed the effects of BC made from different pyrolysis conditions on rapeseed. They reported that slow pyrolysis increased N accumulation in tissues and decreased Cr toxicity, leading to robust growth [120]. This improvement was related to the fact that slow pyrolysis increases the surface area and oxygen function, thereby reducing Cr uptake and its toxic impacts [120].

More importantly, reducing Cr accumulation reduces H_2_O_2_ and MDA production, thereby ensuring better growth [167]. Lalarukh et al. [24] reported that BC made from poultry manure promoted plant growth by increasing antioxidant activity and nutrient availability and decreasing MDA and H_2_O_2_ production. The findings of Al-Farraj et al. [168] revealed that BC substantially enhanced tomato growth, increasing antioxidant activity and decreasing Cr accumulation. Aziz et al. [169] reported that the performance of BC can be increased by the use of BC in combination with microbes. They reported that BC and bacteria (*Bacillus subtilis* and *Pseudomonas aeruginosa*) enhanced maize growth by increasing antioxidant activity, nutrients, and SOC availability [169]. Biochar increases Cr stabilization, which can be quantified through the bioconcentration factor (BCF), bioaccumulation factor (BAF), and bioaccumulation concentration. For example, Naveed et al. [109] reported that BC application to Cr-polluted soil (25 mg kg^−1^) resulted in the lowest BCF (0.43%), BAF (0.0000526%), and BAC (0.1667) values. They reported that BC decreased health risks by decreasing Cr accumulation. The addition of cocomposted BC also reduced daily metal intake (DIM), cancer risk (CR), and the total hazard quotient (THQ). Sami et al. (2023) reported that BC in combination with selenium reduced the health risk index (HRI) by decreasing the accumulation of Cr in plant parts [82]. Moreover, Naveed et al. [109] reported that composted BC also resulted in DIM and HRI values < 1, indicating that Brassica consumption was safe with no HR. Biochar causes the adsorption, reduction, and immobilization of Cr in soil, which reduces Cr uptake and accumulation, hence decreasing the health risk. These findings suggest that BC improves plant growth in Cr-contaminated soils, which is associated with enhanced plant functioning and soil chemical pathways. The improvement from germination to growth and BC is linked with the ability of BC to reduce oxidative damage and enhance nutrient homeostasis, photosynthetic efficiency, plant function, and soil health. Furthermore, these findings indicated that growth improvement largely depends on BC application rates, and 2–5% application rates resulted in promising improvements in growth. Additionally, the BC production method, particularly pyrolysis temperature and modifications, has superior results in reducing Cr toxicity and enhancing crop growth. In addition, BC also decreases Cr accumulation and health risks, indicating that BC could be a viable strategy for enhancing safer crop production in Cr-polluted soils.

## 4. Different Mechanisms Mediated by Biochar to Remove Chromium from Contaminated Environments

The removal of Cr with BC from a toxic environment involves a different mechanism. Adsorption is a primary mechanism involving Cr removal from the environment [170,171,172]. Thangagiri et al. [172] reported that BC removed Cr via surface adsorption, which was confirmed by XPS spectra [173]. Biochar also has large functional groups, with a high cation exchange capacity, which helps in removing Cr from the environment [174]. Biochar also favors ion adsorption to remove Cr from the environment. For example, Li and colleagues reported a reduction in Ca, Mg, and Na ions and suggested that BC-mediated ion exchange promoted the sorption of Cr [175]. Biochar has a positively charged surface area, and it binds toxic metals via electrostatic interactions [176]. An increase in growth medium pH increases the negative charge on BC, which increases the electrostatic attraction with Cr(III) [177]. Furthermore, indirect reduction is also carried out on the BC surface because of electrostatic attraction, where Cr-VI is reduced into Cr-III [178]. The development of a surface complex of Cr with BC is another mechanism used to mitigate Cr toxicity [179]. For example, in different crops, such as canola, rice, peanut, and soybean, BC enhances the adsorption of Cr-III [179]. The primary role of oxygen-containing functional groups on BC is to function as electron donors, which facilitate the reduction of Cr-VI into Cr-III. The subsequent Cr-III cations are then immobilized by surface complexation, cation exchange, and precipitation, thereby decreasing Cr availability in the environment [179].

The ability of biochar to mitigate Cr toxicity is also linked to its ability to facilitate the reduction of highly toxic and mobile Cr-VI to less toxic and immobile Cr-III. Previous studies reported that BC remediates Cr-VI-contaminated soils by transforming Cr-VI into Cr-III [180,181]. Biochar facilitates the transformation of Cr by increasing the soil pH and its uptake by plants [182]. In a separate investigation, Zhu et al. [183] demonstrated that biochar (BC) facilitates the direct reduction of chromium (Cr) through the action of surface-modulatory environmentally persistent free radicals (EPFRs). Similarly, other researchers have observed that EPFRs present on BC are capable of reducing hexavalent chromium (Cr-VI) to trivalent chromium (Cr-III), thereby mitigating its toxic effects [184]. Additionally, BC has been shown to reduce Cr-VI to Cr-III due to the presence of specific functional groups on its surface [185,186]. For example, Zhong et al. [186] identified that hydroxyl (–OH) and amine (–NH_3_) groups significantly contribute to electron donation, facilitating the reduction of Cr-VI. The oxygen-containing functional groups, such as –C-O and –C=O, present on BC act as electron donor moieties of BC for the reduction of Cr-VI [187,188]. Biochar containing oxygenated groups such as −OH and −C=O could directly reduce Cr-VI to Cr-III [189]. Moreover, Cr-III after the reduction of Cr-VI is immobilized by BC owing to the carboxyl groups present on the BC surface. Additionally, BC increases the amount of organic matter in soil, which promotes the reduction of Cr-VI by mediating electron transfer between BC and Cr-VI ions [188,190]. Overall, the prevailing findings suggest that reduction followed by immobilization is the interlinked mechanism responsible for Cr removal; however, the properties of BC determine the dominant pathway. Nevertheless, it is difficult to predict whether Cr absorption by BC consistently precedes reduction or if the dissolved components of BC reduce Cr in soil before adsorption. A critical knowledge gap exists in validating these processes, especially in terms of assessing the long-term stability of immobilized Cr-BC complexes under real-world field conditions. Converting contaminated soils to fertile ones or directly restructuring microbial communities are somewhat general and are largely based on laboratory studies. Therefore, the long-term stability of Cr–BC complexes under field conditions and their applicability across different soils remain unclear.

## 5. Practical Problems and Challenges in Remediating Polluted Soils

Generally, BC is made from industrial waste, plant biomass, residues, and agricultural waste. The origin of the feedstock significantly affects the BC properties. Biochar may contain toxic metals that can affect soil quality and human health; therefore, efforts are needed to optimize BC production [26]. In addition to toxic metals, BC also contains dioxins, volatile compounds, and polycyclic aromatic hydrocarbons, which are serious threats to humans. Thus, great care must be taken to select the feedstock for producing BC. Biochar also contains toxic metals; therefore, it can increase the availability of toxic metals and subsequent accumulation in plants [28]. Yao et al. [191] reported that BC application in saline agriculture affected the nitrification process. Biochar application increased SOC and altered NO_3_^−^-N availability [191]. This created a suboptimal growth environment and led to inhibited growth of ammonia-oxidizing microorganisms. Xiang et al. [28] reported that BC contains polycyclic aromatic hydrocarbons (PAHs), which are absorbed by plants and lead to a reduction in plant growth. Studies on Pak choi and cabbage have shown that BC application increases the accumulation of PAHs in these plants, exceeding the maximum allowable levels. Liu et al. [192] reported that toxic metals released from maize straw BC inhibited soil microbial activity, and Wang et al. [193] reported that BC from sawdust and rice husks had negligible effects on soil microbes. These findings underscore that the concentration and type of pollutants present in BC lead to differing toxicities.

**Figure 3:**
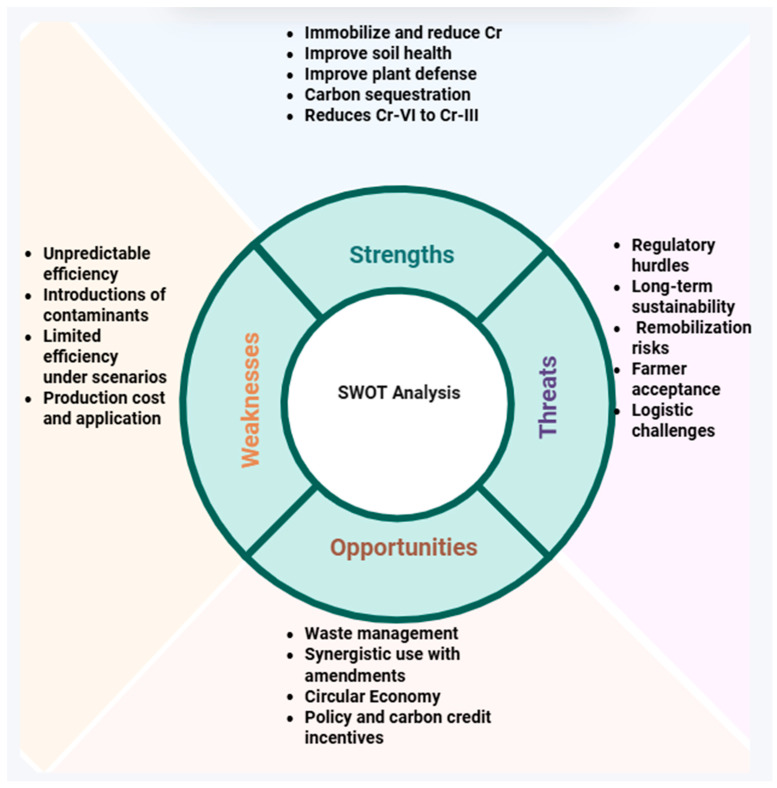
Strengths, weaknesses, opportunities and threats of biochar in remediating Cr-polluted soils and mitigating its toxicity on plants.

In addition, the use of BC in agricultural soils presents many challenges. The primary hurdle is economic and logistical issues, along with the production of high-quality BC for practical use. The transportation of low-density materials can also prohibit their widespread use (Figure 3). The effects of BC are not uniform and largely depend on the feedstock type and the conditions used in BC production. Thus, BC could be beneficial for one soil type but may be detrimental for other soil types, potentially changing the soil pH and immobilizing essential nutrients. The long-term behavior of BC in soils has not yet been defined, particularly its stability and interaction with soil microbes. Biochar also has a relatively low density and can float on the water surface, leading to BC loss; thus, it can be used in combination with other amendments.

## 6. Conclusions and Future Prospective

Chromium stress inhibits plant growth by impairing plant functions and decreasing nutrient and water availability. The fact that the growth of crops in Cr-contaminated soils can lead to their entry into humans has serious health implications. Biochar application is a promising strategy to decrease Cr toxicity and availability, hence increasing crop productivity. Biochar works as an adsorbent and dynamic soil amendment, which orchestrates an interplay of complicated mechanisms, reducing Cr availability and stress resistance. Biochar decreases Cr availability through immobilization by complexation, adsorption, and precipitation. Biochar also increases antioxidant activity, photosynthetic recovery, osmolyte synthesis, hormonal balance, and nutrient and water uptake and reshapes the soil bacterial communities, contributing to better growth in Cr-polluted soils. Notably, BC also decreases Cr uptake by plants, thus decreasing the health risk by decreasing Cr entry into the human food chain. The efficiency of BC in mediating these mechanisms is largely influenced by the feedstock type, production conditions, and modification processes. Studies have shown that BC produced at low temperatures has more functional groups that mediate Cr-VI reduction into Cr-III than BC produced at higher temperatures. However, studies are needed to understand the diverse mechanisms involved in counteracting Cr toxicity.

➢ Biochar promotes nutrient uptake; however, the underlying mechanisms are unknown. Therefore, studies are needed to understand these mechanisms. The effects of BC on the anatomical features of plants growing in Cr-polluted soils are known; therefore, understanding these mechanisms can provide better insights for mitigating Cr toxicity.
➢ Future research should develop models linking BC properties with specific goals of reducing Cr. There is also a need to explore the “black box” of plant molecular mechanisms mediated by BC to counteract Cr toxicity. These findings help elucidate the signaling mechanism by which BC affects the expression of genes associated with hormones, antioxidant defense, and nutrient transport. Likewise, the role of BC in soil microbes in Cr-polluted soils has been poorly studied; therefore, advanced omics can be used to explore its role.
➢ Long-term field trials are needed to verify the stability of Cr immobilized by BC. This can help in the development of measures to increase crop productivity in Cr-polluted soils.
➢ Studies are also needed to prioritize field validations under different climate conditions to assess economic benefits and real-world efficiency. Furthermore, cost–benefit and life cycle analyses are crucial for comparing both the sustainability and practicality of simple and modified BC.
➢ The input of secondary contaminants, particularly from modified BC, must be investigated to ensure environmental safety. Converting contaminated soils to fertile ones or directly restructuring microbial communities are somewhat general and are largely based on laboratory studies. The long-term stability of Cr–BC complexes under field conditions and their applicability across different soils remain unclear. Therefore, future studies should aim to explore these interactions.
➢ The integration of BC with other amendments, such as microbes, nanoparticles, compost, and hormones, can create powerful strategies for the remediation of Cr-polluted soils. The source of feedstock from municipal wastes and agricultural waste should be optimized to align BC production with the principles of the circular economy, converting burden to opportunity. This can help remediate Cr-polluted soil, safeguard human health, and ensure safer and sustainable crop production.

## Figures and Tables

**Table 1:** Toxic effects of chromium on plant physiological and biochemical functioning, growth and yield.

Plant Type	Chromium Stress	Growth Media	Effects on Plant Functioning	References
Maize	100 μM for seven days	Hydroponic	Cr decreased the plant growth by increasing O_2_^•−^, H_2_O_2_, and MDA production.	[49]
Chickpea	130 and 260 µM two times per week	Soil	Cr decreased, growth, minerals acquisition, and photosynthetic pigments by increasing ROS production.	[50]
Tomato	50 µM for two weeks	Hydroponic	Cr decreased plant growth by increasing H_2_O_2_, MDA, and EL production.	[51]
Faba bean	300 μM two days per week	Soil	Cr decreased faba bean growth and biomass by inhibiting photosynthesis and increasing MDA (216.11%), H_2_O_2_ (230.16%), EL (293.30%) production.	[52]
Soybean	100 µM with a nutrient media solution for 7 days	Hydroponic	Cr reduced the soybean growth and yield by decreasing germination, chlorophyll synthesis, nutrient uptake and increasing oxidative damages.	[53]
Sunflower	250 mg kg^−1^	Soil	Chromium stress decreased photosynthesis, plant growth, and increased dopamine secretion in rhizosphere.	[54]
Mungbean	250 mg kg^−1^	Soil	Chromium decreased growth by increasing H_2_O_2_ production and Cr accumulation in plant parts.	[55]
Peach	100 and 150 mg kg^−1^	Soil	Cr stress inhibited the chlorophyll content, increased MDA production, Moreover, Cr first increased CAT, POD, and SOD activities and then decreased.	[16]
Black cumin	1.5–4 mM L^−1^	Soil	Notably, 4 mM Cr decreased the chlorophyll (67%) synthesis, and seed yield (43–71%), while increased Cr contents in seed surpassing WHO threshold level of 1.5 mg kg^−1^.	[56]
Maize	100 and 300 µM for 5 weeks	Soil	Cr stress decreased plant height, leaf area, chlorophyll synthesis, and increased H_2_O_2_, MDA, and EL production.	[57]
Spinach	50 and 100 mg kg^−1^	Soil	Cr decreased the growth and biomass and enhanced the SOD, and CAT activities.	[58]
Mungbean	300, and 400 mg kg^−1^	Soil	Cr stress decreased growth rate (82.34%), by decreasing chlorophyll synthesis, APX, CAT, POD and SOD activity.	[59]
Wheat	300, and 600 mg kg^−1^	Soil	A marked reduction in growth and photosynthetic traits were caused by Cr.	[60]
Mint	10–60 mg kg^−1^	Soil	Notably, Cr stress (60 mg kg^−1^) decreased the plant height, (42.8%), plant fresh weight (40.9%), and herbage yield (26.6%) by decreasing photosynthetic pigments and increasing Cr accumulation and oxidative damages.	[61]
Rice	50–400 μM for seven days	Hydroponic	Cr stress decreased seed germination, shoot length, biomass production, carotenoids, photosynthetic rate, transpiration rate, by increasing H_2_O_2_, MDA, and EL production.	[62]
Maize	50 mg kg^−1^	Soil	Cr decreased plant height, biomass production and nutrients accumulation in maize seedlings.	[63]
Rice	100 μM	Soil	Cr stress decreased the rice growth and productivity by decreasing gas exchange traits, and oxidative stress biomarkers.	[64]
Maize	100 mg L^−1^	Soil	Cr reduced the germination attributes, biomass production by decreasing chlorophyll contents and increasing oxidative damages.	[65]
Maize	100 and 500 µM	Soil	Cr stress significantly decreased gas exchange attributes, nonenzymatic compounds, plant growth and increased oxidative damages.	[66]
Tomato	100 and 500 mg L^−1^	Soil	Notable 500 mg L^−1^ decreased the germination (41.9%), growth, and activity of *Escherichia coli*, Agrobacterium rhizogenes, and *Agrobacterium tumefaciens*.	[67]

**Table 2:** Role of biochar in mitigating chromium stress in modulating plant functioning, chromium availability and soil properties.

Plant Type	Chromium Stress	Growth Media	Biochar Application	Effects on Plant Functioning	References
Rice	300 mg kg^−1^	Soil	1%	Biochar addition increased the root biomass (23–65%) and decreased root Cr contents (46–74%) by increasing soil organic matter availability, organic matter contents.	[89]
Brassica	20 mg L^−1^	Hydroponic	0.5–2.5 g L^−1^	Biochar supply (2.5 mg L^−1^) enhanced the plant growth, mitigated the oxidative damages by increasing soluble sugars (52.8%), protein contents (114.4%), and decreasing Cr accumulation.	[90]
Bottle gourd	100 mg kg^−1^	Soil	2%	Biochar increased vine length, fresh and dry biomass, chlorophyll synthesis and membrane stability.	[91]
Wheat	5, 10, 20 and 40 mg L^−1^	Petri dish	0.2 g per dish	Biochar enhanced plant dry biomass (250%) and decreased the Cr accumulation.	[92]
Maize	600 mg kg^−1^	Soil	1.0, 2.5, 5.0 and 10%	Biochar enhanced maize growth by increasing, soil pH, organic matter and nutrients availability, and transforming Cr(III) into Cr(VI).	[93]
Maize	5 and 150 mg kg^−1^	Soil	0.5, 1, 1.5, and 2%	Biochar (2%) addition increased root biomass (99.7%), grain yield (98.2%), by increasing chlorophyll synthesis, antioxidants activity and decrease Cr accumulation in roots and shoots.	[94]
Maize	20 mg kg^−1^	Soil	0.50 mg kg^−1^	Biochar supply increased root length (23%), shoot length (23%), by increasing POD (40%), CAT (41%) activity and decreasing the Cr accumulation.	[95]
Mungbean	25 mg kg^−1^	Soil	5%	Biochar enhanced the germination, plant biomass, photosynthetic pigments, and antioxidants activities (CAT, POD and SOD).	[96]
Maize	255 mg kg^−1^	Soil	4500 kg ha^−1^	Biochar enhanced the maize growth and yield and by decreasing Cr accumulation.	[97]
Wheat	75 and 150 mg kg^−1^	Soil	20 g kg^−1^	Biochar plant growth by increasing chlorophyll-a (155%), chlorophyll-b (41%), proline (60.2%), phenolics (96.4%) synthesis and decreasing Cr accumulation.	[98]
Wheat	50, 100 and 200 mg kg^−1^	Soil	10%	Biochar decreased the MDA production, by increasing POD, CAT, SOD and H_2_O_2_ activities.	[74]
Tomato	0.25 mM	Soil	10 g kg^−1^ soil	Biochar mitigated the Cr toxicity by decreasing H_2_O_2_, MDA, EL production and increasing antioxidants gene expression.	[99]
Basil	429.85 mg kg^−1^	Soil	0.5%	Biochar improved soil quality by decreasing Cr uptake and increasing Mg, and Fe concentration in plants tissues	[100]
Brassica	100 and 200 mg kg^−1^	Soil	30 g kg^−1^ soil	Biochar increased soil pH, nutrient uptake and reduced the Cr availability and its absorption.	[101]
*Ricinus communis*	150 mg kg^−1^	Soil	20 g kg^−1^ soil	Biochar enhanced plant growth, soil quality index, and relative abundance of *Arthrobacteria*.	[102]
Sunflower	14.34 mg kg^−1^	Soil	8.0 t ha^−1^	Biochar addition decreased the Cr toxicity and enhanced yield and yield traits, nutrient availability, and seed quality.	[103]
Lavender	50 mg kg^−1^	Soil	30 g kg^−1^ soil	Biochar decreased Cr accumulation in leaves (39–60%), increased soil nutrient availability, photosynthetic pigments, IAA synthesis (15–29%) synthesis and decreased synthesis of jasmonic acid (4–17%), salicylic acid (29–49%), and abscisic acid (30–66%).	[104]
Tobacco	150 mg kg^−1^	Soil	4 g kg^−1^ soil	Biochar application decreased extractable Cr in soils by increasing the Cr adsorption, and soil enzymes activity.	[105]

## Data Availability

No new data were created or analyzed in this study. Data sharing is not applicable to this article.
